# The impact of early life factors on cognitive function in old age: The Hordaland Health Study (HUSK)

**DOI:** 10.1186/2050-7283-1-16

**Published:** 2013-09-16

**Authors:** Jens Christoffer Skogen, Simon Øverland, A David Smith, Arnstein Mykletun, Robert Stewart

**Affiliations:** Faculty of Psychology, Department of Health Promotion and Development, University of Bergen, Bergen, Norway; Division of Mental Health, Department of Public Mental Health, Norwegian Institute of Public Health, Bergen, Norway; Department of Pharmacology, University of Oxford, Oxford, UK; King’s College London (Institute of Psychiatry), London, UK

**Keywords:** Early life factors, Old age, Cognitive function, Risk factors

## Abstract

**Background:**

Previous studies have shown that adverse conditions during fetal and early life are associated with lower performance on neurocognitive tests in childhood, adolescence and adult life. There is, however, a paucity in studies investigating these associations into old age. The aim was to investigate the impact of early life factors on cognitive function in old age by taking advantage of the potential for a linkage between a community survey and historical birth records.

**Methods:**

A historical cohort study employing a linkage between a community survey of people aged 72–74 years with the participants’ birth records (n=346). Early life factors included anthropometric measures taken at birth, birth complications, parental socioeconomic status, and maternal health status. The main outcome was a z-scored composite cognitive score, based on test scores from Kendrick Object Learning Test, Trail Making Test A, a modified version of the Digit Symbol Test, Block Design, a modified version of Mini-Mental State Examination and an abridged version of the Controlled Oral Word Association Test (COWAT). The separate cognitive tests were also individually analysed in relation to measures identified at birth.

**Results:**

Higher parental socioeconomic status (SES; based on father’s occupation) was associated with a higher value on the composite cognitive score (by 0.25 SD, p=0.0146) and higher Digit Symbol and Trail Making Test A performance. Higher head circumference at birth was associated with higher COWAT and Trail Making Test A performance. Both higher parental SES and head circumference at birth predicted cognitive function in old age independently of each other. There were no other consistent associations.

**Conclusions:**

In general we found little evidence for a substantial role of early life factors on late-life cognitive function. However, there was some evidence for an association with parental SES status and head circumference on certain cognitive domains.

## Background

Age-associated cognitive decline and mild cognitive impairment in old age is a major public health challenge (Deary et al. 2009), with the steady increase in life expectancy seen worldwide (National Institute on Aging 2007). A recent review estimated the prevalence rates of mild cognitive impairment to be in the range of 14% to 18% for individuals aged 70 years or more (Petersen et al. 2009), with the prevalence increasing as a function of age (Golomb et al. 2004). It is difficult to distinguish non-pathological and pathological cognitive problems in old age (Deary et al. 2009), but both cognitive decline and impairment are associated with lower quality of life, increased disability and neuropsychiatric symptoms, as well as being associated with higher risk of later dementia and mortality (Deary et al. 2009; Lyketsos et al. 2002; Bierman et al. 2007).

Cognitive function in later life is associated with factors manifesting across the life course such as mid-life cardiovascular and metabolic factors (Breteler et al. 1994; Gatto et al. 2009), but also early life factors including educational attainment (Cagney & Lauderdale 2002) and skeletal growth (Mak et al. 2006). Associations have been found between lower birth weight and a range of later adult outcomes including ischemic heart disease, hypertension, obesity and diabetes (Barker 1995; Hales & Barker 2001; Barker et al. 1993; Barker 2004), and given the close relationship between these conditions and cognitive function, a potential link exists between foetal development and later cognitive deficits (Whalley et al. 2006). The long-term effects between early life-factors and outcomes later in the life-course are conceptually referred to as the “fetal origins of adult disease” and have been thought of as an essential shift in our understanding of determinants for health (Skogen & Øverland 2012). From a public health perspective, the possible link between early life factors and late life function and disease, may inform our thinking about when and how to prevent and intervene (Skogen & Øverland 2012; Kajantie 2008).

Several studies have found that lower birth weight is associated with later lower intellectual abilities (IQ) and lower performance on tests of neurocognitive function in childhood, adolescence and adult life (Sorensen et al. 1997; Shenkin et al. 2001; Richards et al. 2002; Lundgren et al. 2003; Jefferis et al. 2002). This has not only been shown in follow-up studies of children born premature or small for gestational age (Lundgren et al. 2003), but also for birth weight within normal ranges (Jefferis et al. 2002). Length at birth has also been found to be associated with later intellectual performance (Lundgren et al. 2003). However, despite the potential link of the early life environment with later function as well as with metabolic and cardiovascular risk factors, to our knowledge only three studies have investigated the association between foetal development and cognition in more advanced age for both genders (Martyn et al. 1996; Gale et al. 2003; Zhang et al. 2009). Two found no evidence for this (Martyn et al. 1996; Gale et al. 2003), while the third found that most prenatal factors were associated with cognitive function in old age in unadjusted models (Zhang et al. 2009), but these associations were substantially attenuated by adjustment for intervening lifespan factors (Zhang et al. 2009). Cohorts with data on both early- and late-life environment are rare, and all three previous studies were limited in the number of relevant exposures (Gale et al. 2003), and assessment of cognitive function (Martyn et al. 1996; Gale et al. 2003), as well as in their age range (the third study having participants aged 50–82 years, but most of whom were in the 50–58 year range). Further research is therefore needed (Erickson et al. 2010), and to the best of our knowledge no studies have investigated these issues among community-dwelling individuals over 70 years of age.

Employing a unique linkage between a community survey and a historical birth record archive, we were able to investigate a range of early life factors in relation to cognitive function on a battery of assessments in community residents aged 72 to 74 years. Specifically, we investigated the prospective association between anthropometric measures taken at birth, birth complications, parental socioeconomic status, and maternal health status in relation to scores on a cognitive test battery in old age.

## Methods

### Study population

**Figure 1:**
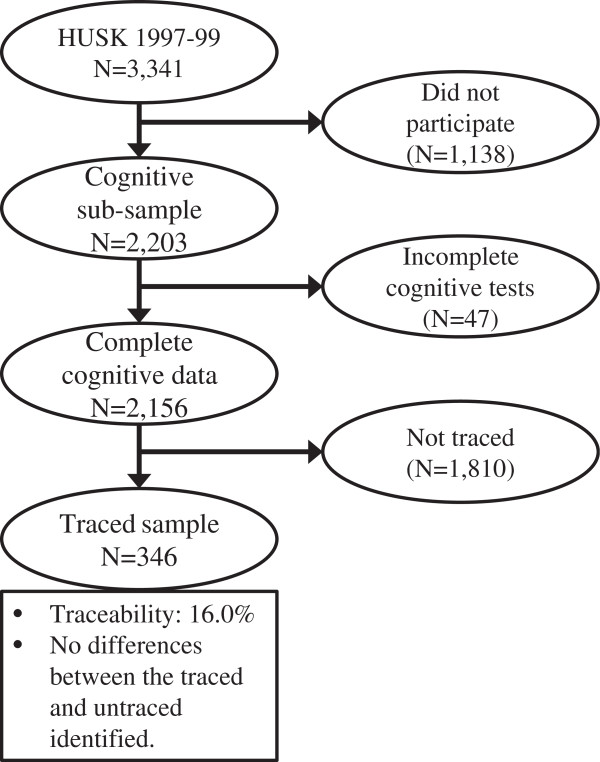
**Flowchart describing the establishing of the final study population.**

The sampling frame for this study comprised the participants of the old age cohort of the population-based Hordaland Health Study (HUSK) which has been described in more detail elsewhere (Refsum et al. 2006). In summary, all residents of Bergen city or neighbouring areas born during the period of 1925–27 of a previously established cohort were invited to participate in a general physical examination and to complete a set of questionnaires on socio-demographic status, general health and health-related behaviour. HUSK was conducted from 1997 to 1999 as a collaboration between the National Health Screening Service, the University of Bergen and the local health services. A random subsample of the attendees in the old age cohort (n=3,341) was also invited to participate in a cognitive examination, with 2,203 (66% of the attendees) agreeing to participate (Figure 1). Of these, 2,156 had complete data and were included in analyses presented here.

In the Norwegian Population Registry, all inhabitants of Norway are registered with a personal identification number. Using this individual identifier, the names (and maiden name for females), date of birth, place of birth and parents’ names (when available) of HUSK participants were retrieved. This information was used to trace the participants born in Bergen to the birth records from the public maternity ward (“Fødestiftelsen i Bergen”) presently stored at the Regional State Archives of Bergen. In the second decade of the 20th century, about one quarter of all births in the Bergen area took place in the official maternity ward (personal communication, State archivist). The proportion of deliveries taking place at hospitals increased steeply when the new Women’s Clinic (“Kvinneklinikken”) was inaugurated in 1926, replacing the old maternity ward. The pertinent birth records for the present study were those detailing births between 1st of January 1925 and 31st of December 1927, and these records have been employed previously in a similar study design (Skogen et al. 2013). The records contain detailed information about the pregnancy, the birth process and the mother’s health recorded by midwives and obstetricians during the hospital stay. The Women’s Clinic in question was the main teaching facility for midwifes at the time, and the records were requisite for the training, and are therefore considered to be of high quality (Rosenberg 1987). Of the 2,156 participants in the HUSK cognitive examination, we were able to trace 346, which constituted the final study sample aged 72–74 years (mean 72.3).

### Early life factors – information obtained at birth, 1925–27

The available birth records in the Regional State Archives of Bergen were viewed and coded blind to all HUSK measures. The following information was abstracted from the record (directly copying original information unless stated otherwise): birth weight (kg), birth length (cm), head circumference (cm) at birth, ponderal index (PI; calculated from weight and length), mother’s pelvic size (the mean of the interspinous distance, the intercristal distance and the external conjugate in centimeters). The following binary variables were derived from individual free text fields: any recorded disease in the mother (yes/no), family history of coronary heart disease (yes/no) and tuberculosis (TB; yes/no), the state of mother’s teeth (poor/good), mother’s condition after birth (poor/good), complications during birth (including, but not limitied to, prolonged labour, abnormal presentation, assisted delivery of the baby (use of forceps) and episiotomy, uterine rupture, discoloured amniotic fluid, abnormal fetal souffle and placenta praevia; yes/no), mother’s general somatic state at discharge (poor/good), marital status (married/unmarried), socioeconomic status (based on father’s occupation; lower/higher), and type of payment for the hospital stay (health insurance/other).

### Cognitive examination at age 72–74 years of age

HUSK included a cognitive test battery consisting of six tests. The cognitive tests are in wide use internationally and have been well validated, including the Norwegian versions of MMSE and KOLT (Kendrick 1985; Wechsler 1981; Benton & Hamscher 1989; Braekhus et al. 1992; Reitan 1958; Engedal et al. 1988). Two assessors were trained over two days to use the test battery (personal communication, Professor Knut Engedal). These assessors were nurses, and the battery was administered on-site by the trained nurses at the end of the study’s examination.

#### ***Kendrick object learning test (KOLT)***

The Kendrick Object Learning Test is designed to assess episodic memory performance (Kendrick 1985). The maximum score of KOLT is 70, and the range in our study sample was 6–60.

#### ***Trail making test a (TMA)***

The Trail Making Test A is a test of visual conceptual and visuomotor tracking (Reitan 1958). The test involves both motor speed and attention functions. The score is equivalent to the time in seconds to complete the items, and was between 16–154 seconds in our study sample. For TMA we reversed the scale to ensure that high and low scores corresponded with the other tests.

#### ***Modified version of the digit symbol test (digit symbol)***

The modified version of the Digit Symbol Test measures perceptual and psychomotor speed, focused attention and visuomotor coordination (Wechsler 1981). In the version administered, the number of correct matches between digits and symbols in 30 seconds was recorded. The range in our study sample was 2–22.

#### ***Block design***

The Block Design test investigates visuospatial and motor skills (Wechsler 1981). In the current version 4 of the 10 patterns (pattern 1, 2, 5 and 6) from the full study was included. The maximum score was 16 in this short form. The range in our study sample was 2–16.

#### ***Modified version of the mini-mental state examination (MMS)***

The Modified version of the Mini-Mental State Examination is designed to test various aspects of cognitive function, including orientation, instant recall and memory (Braekhus et al. 1992). It involves orientation to time and place, naming, repeating, writing, copying, immediate recall, delayed recall, backward spelling, and performing a 3-stage oral instruction. The modified version consists of 12 of the 20 items of the full version and has been shown to be similar in the ability to identify cognitive impairment in the elderly (Braekhus et al. 1992). The range of scores in our study sample was 5–12.

#### ***Abridged version of the controlled oral word association test (COWAT)***

The abridged version of the Controlled Oral Word Association Test assesses semantic memory, verbal fluency and psychomotor speed (Benton & Hamscher 1989). The subjects were required to generate as many words as possible beginning with the letter “S” within 60 s. The range in our study sample was 3–34.

Based on these tests a Z-scored (standardized to a mean of 0 and standard deviation of 1) composite cognitive scale was constructed by summing the separate standardized scores for each of the tests. The composite cognitive score constitutes the main outcome in this study.

### ***Context for the birth cohort***

During the late 19th century and early 20th century, Bergen city expanded geographically, and went from a semi-rural city to a city with more modern characteristics. Primary industry which had dominated gave way for an expanding secondary and tertiary industry (Ertresvaag 1982). This change in industry was mostly due to growing production and manufacturing, but also due to an increase in commerce, shipping and transport, and service sector (Ertresvaag 1982). As a consequence of this, three social classes began to dominate in Bergen during the same period, upper (bourgeoisie), middle and lower, with large differences in income, housing standard and diet. The upper class was characterised by financers, importers, industry proprietors and wholesale dealers. The middle class consisted primarily of craftsmen, merchants and officials, while the lower class comprised regular worker or artisans (Ertresvaag 1982). During 1925 and 1927 the life expectancy in Norway was approximately 67 years for males, and 74 years for females (Mamelund & Borgan 1996).

### Additional information gathered during follow-up from HUSK at age 72–74

Potential differences in the distribution of gender, self-reported level of educational attainment and general health were investigated between the HUSK participants with birth journal information (N=346) and participants without (N=1,810). Level of educational attainment was divided into “compulsory only” (up to ten years) and “post-compulsory” (11 years or more), while general health was divided into “poor” and “good”. As APOE gentotype has been associated with cognitive function (Izaks et al. 2011), information about apoE4-status (presence of any E4-allele versus absence of E4-allele) was also included (using nonfasting plasma samples taken during the general physical examination of HUSK).

### Statistical analyses

HUSK participants with traceable birth records were compared to the remainder of the HUSK participants. Bivariate and age- and gender-adjusted associations were then investigated between exposures and outcomes employing linear regression models. Our approach was to investigate and report all associations between exposures and outcomes, taking into account the number of significant associations that would be expected through chance alone, but also evaluating the output for any consistency in associations for a given exposure or outcome (Rothman 1990). For the main analysis, Stata version 11.0 (StataCorp 2010) was employed. Using the software G*Power version 3.1.3 (http://www.psycho.uni-duesseldorf.de/abteilungen/aap/gpower3/) a power analysis indicated that we would be able to detect a small to medium effect size for continuous outcomes (a correlation of 0.13), and mean differences (Cohen’s d of 0.35) at a power of 80% (alpha 0.05) given our sample size (Cohen 1992). We also investigated the potential two-way interaction between apoE4-status and gender for each of the exposures in relation to the composite score, in a post-hoc analysis. Post-hoc analyses were also performed to investigate whether the effect of parental SES on cognitive function were independent of anthropometric measures, as well as whether the effect of head circumference on cognitive function was independent of parental SES. In sensitivity analyses, we also explored the effect of separate additional adjustment for educational attainment and self-rated general health on those associations found to be significant after age- and gender-adjustment.

### Ethics

The data in HUSK was collected in accordance with ethical standards required by the regional ethical board of Committees for Medical and Health Research Ethics in Norway (REC). The permission to collect and store the data from HUSK was given by the Norwegian Data Inspectorate. All participation in HUSK was voluntary, and all potential participants received written information about the project before they met for examination. The participants gave their written statements of informed consent, including the specific consents to use information from HUSK in health research and to link this information with other relevant data sources. This specific study was reviewed and approved by REC.

## Results

No systematic differences were found between the HUSK participants we were able to trace, compared to the rest of the participants with regards to gender, educational attainment, self-reported health, or the cognitive tests (Table 1). The sample characteristics of the analysed sample are summarized in Table 2.

**Table 1:** **Differences on demographics and outcomes between HUSK-participants with birth journal information (N=346) and participants without (N=1,810)**

		Proportion/mean with birth journal information	Proportion/mean without birth journal information	p-value/Mean difference (CI95%)*
Gender (% female)		53.8%	55.3%	p=0.599
Education (% post-compulsory)		60.5%^a^	62.9%^b^	p=0.429
Self-reported health (% good)		70.5%^c^	67.6%^d^	p=0.311
Composite score^e^		0.08	−0.01	0.09 (−0.02, 0.21)
*Separate cognitive tests* ^f^				
	MMS	11.55	11.51	0.04 (−0.05, 0.13)
	Digit Symbol	10.60	10.18	0.42 (−0.06, 0.91)
	KOLT	35.49	35.17	0.32 (−0.61, 1.25)
	COWAT	15.51	15.02	0.49 (−0.15, 1.12)
	TMA	55.23	57.67	−2.44(−6.39, 1.50)
	Block Design	15.01	15.01	0.00 (−0.26, 0.26)

95% confidence intervals in brackets.

*p-values derived from χ^2^ and mean difference derived from independent samples t-tests.

^a^Available information from N=326.

^b^Available information from N=1655.

^c^Available information from N=335.

^d^Available information from N=1789.

^e^Composite score: Z-score of the sum of all cognitive tests (mean: 0, standard deviation: 1).

^f^Test-specific raw scores for each cognitive tests (see Methods section for further details).

**Table 2:** **Sample characteristics at birth obtained from medical records, and at age 72–74 years obtained from HUSK**

		N	Mean (SD*)	Proportion (%)
**From medical records**				
Birth weight (kg)		346	3.47 (0.53)	-
Birth length (cm)		346	50.27 (2.11)	-
Head circumference (cm)		344	34.42 (1.70)	-
Ponderal index (weight/height^3^)		346	2.64 (0.24)	-
Mother’s pelvic size (cm)^a^		324	26.10 (1.33)	-
Mother’s age		346	29.40 (5.85)	-
Parity (number of births)		346	2.61 (1.95)	-
Gender (% female)		346	-	53.8%
Mother’s condition after birth (% good)		346	-	90.2%
Tuberculosis in family (% no)		346	-	88.7%
CVD in family (% no)		346	-	87.0%
Mother’s appearance (% good)		346	-	76.3%
Complications birth (% no)^b^		346	-	89.6%
Socioeconomic status (% lower)		346	-	55.8%
Unmarried (% no)		346	-	96.2%
Teeth lower jaw (% good)		335	-	42.1%
Type of payment (% no insurance)		245	-	44.1%
Number of diseases (%≤1)		346	-	69.4%
**From HUSK at age 72–74 years**				
Composite score^c^		346	0.08 (0.95)	-
*Separate cognitive test* ^d^				
	MMS	346	11.55 (0.85)	-
	Digit Symbol	346	10.60 (4.37)	-
	KOLT	346	35.49 (7.86)	-
	COWAT	346	15.51 (5.40)	-
	TMA	346	55.23 (29.06)	-
	Block Design	346	15.01 (2.08)	-

*Standard deviation.

^a^Mean of the interspinous distance, the intercristal distance and the external conjugate in centimeters.

^b^Including, but not limited to, prolonged labour, abnormal presentation assisted delivery of the baby (use of forceps) and episiotomy, uterine rupture, discoloured amniotic fluid, abnormal fetal souffle and placenta praevia, and combinations of these.

^c^Composite score: Z-score of the sum of all cognitive tests (mean: 0, standard deviation: 1).

^d^Test-specific raw scores for each cognitive tests (see methods section for further details).

Out of the 136 crude associations investigated, only 10 (7.4%) were significant at α=0.05 (Tables 3 and 4), and in general there were few patterns or consistencies observed among these significant associations. Head circumference was positively associated with COWAT and TMA performance but not with the composite score (Table 3). Parental SES was the only exposure that was associated with the composite score, where a higher parental SES was associated with an increased mean score by 0.25 standard deviation (p=0.0146). A higher parental SES was also associated with a better Digit Symbol and TMA performance (Table 4). Adjusting for age and gender rendered the association between head circumference and TMA performance non-significant, but the other significant associations were unaltered (Table 3). None of the other significant associations in our sample were consistent or indicative of any specific pattern. There was no evidence for interaction between apoE4-status and any exposures in relation to the composite cognitive score (p-values for interaction term ranging from 0.190 to 0.866). We found a significant interaction between gender and the reported condition of the mother’s teeth (p=0.008) with an exploratory gender-stratified analysis indicating that reported poor dentition in the mother was associated with a worse composite cognitive function score in old age, but for female participants only (mean difference 0.34, p=0.014). In a post-hoc analysis, a higher parental SES predicted a higher cognitive function in old age independently of birth anthropometric measures, and head circumference predicted some aspects of cognitive function in old age independently of parental SES. The results of these post-hoc analyses were analogous to the age- and gender-adjusted models (data not shown). For the significant age- and gender-adjusted associations identified, we carried out additional separate adjustments for educational attainment and self-rated general health. Adjusting for self-rated health only slightly affected the associations, while adjusting for educational attainment affected some of the associations to a larger degree. Specifically, the associations between paternal SES and cognitive function were substantially weakened (about 60-80% reduction in effect sizes of point-estimates).

**Table 3:** **Associations between continuous individual risk factors at birth and continuous cognitive outcomes at age 72–74 years (N=346)**

Exposures	Level of adjustment	Composite score^a^	Separate cognitive tests^b^					
			MMS	Digit symbol	KOLT	COWAT	TMA	Block design
Birth weight (kg)								
	Unadjusted	0.01 (−0.18,0.20)	−0.03 (−0.20,0.14)	−0.12 (−0.99,0.75)	−0.24 (−1.80,1.31)	0.85 (−0.22,1.92)	2.44 (−3.32,8.21)	−0.23 (−0.64,0.19)
	+ age/gender	0.02 (−0.17,0.21)	−0.03 (−0.20,0.14)	−0.14 (−1.02,0.73)	0.24 (−1.29,1.78)	0.91 (−0.18,1.99)	2.01 (−3.81,7.84)	−0.26 (−0.68,0.16)
Birth length (cm)								
	Unadjusted	0.02 (−0.03,0.07)	0.01 (−0.03,0.05)	0.03 (−0.19,0.25)	0.01 (−0.38,0.41)	0.20 (−0.07,0.47)	1.00 (−0.46,2.46)	−0.04 (−0.14,0.07)
	+ age/gender	0.03 (−0.02,0.07)	0.01 (−0.03,0.05)	0.03 (−0.20,0.25)	0.22 (−0.17,0.61)	0.23 (−0.04,0.51)	0.94 (−0.56,2.43)	−0.04 (−0.15,0.06)
Head circumference^c^ (cm)								
	Unadjusted	0.05 (−0.01,0.11)	0.04 (−0.01,0.09)	−0.06 (−0.33,0.22)	−0.05 (−0.54,0.43)	**0.48** ^**^ **(0.15,0.82)**	**1.95** ^*^ **(0.15,3.76)**	0.05 (−0.08,0.18)
	+ age/gender	0.06 (−0.00,0.12)	0.04 (−0.01,0.09)	−0.07 (−0.35,0.21)	0.17 (−0.31,0.65)	**0.52** ^**^ **(0.18,0.86)**	1.81 (−0.04,3.66)	0.04 (−0.09,0.18)
Ponderal index (weight/height^3^)								
	Unadjusted	−0.11 (−0.54,0.31)	−0.17 (−0.55,0.22)	−0.62 (−2.59,1.34)	−0.64 (−4.17,2.89)	1.15 (−1.28,3.57)	−2.87 (−15.91,10.17)	−0.24 (−1.17,0.70)
	+ age/gender	−0.16 (−0.59,0.27)	−0.19 (−0.58,0.19)	−0.74 (−2.73,1.24)	−1.38 (−4.83,2.07)	1.08 (−1.37,3.53)	−3.34 (−16.49,9.82)	−0.27 (−1.21,0.68)
Mother’s pelvic size^d, e^ (cm)								
	Unadjusted	0.06 (−0.02,0.13)	**0.08** ^*^ **(0.01,0.15**)	0.16 (−0.20,0.53)	0.18 (−0.48,0.83)	0.38 (−0.06,0.82)	1.00 (−1.41,3.42)	−0.12 (−0.29,0.06)
	+ age/gender	0.05 (−0.03,0.13)	**0.08** ^*^ **(0.00,0.15)**	0.14 (−0.23,0.51)	0.22 (−0.43,0.86)	0.39 (−0.06,0.83)	0.75 (−1.70,3.21)	−0.14 (−0.32,0.03)
Mother’s age (years)								
	Unadjusted	0.01 (−0.01,0.02)	0.01 (−0.00,0.03)	0.02 (−0.06,0.10)	0.04 (−0.10,0.18)	−0.01 (−0.11,0.09)	−0.19 (−0.71,0.34)	0.00 (−0.04,0.04)
	+ age/gender	0.00 (−0.01,0.02)	0.01 (−0.00,0.03)	0.02 (−0.06,0.10)	0.02 (−0.12,0.16)	−0.01 (−0.11,0.09)	−0.16 (−0.69,0.36)	0.00 (−0.04,0.04)
Parity (number of births)								
	Unadjusted	−0.02 (−0.07,0.03)	0.03 (−0.02,0.07)	−0.14 (−0.37,0.10)	0.12 (−0.31,0.55)	−0.10 (−0.39,0.19)	−1.01 (−2.59,0.57)	−0.11 (−0.22,0.01)
	+ age/gender	−0.02 (−0.07,0.03)	0.02 (−0.02,0.07)	−0.14 (−0.38,0.10)	0.05 (−0.37,0.47)	−0.11 (−0.40,0.19)	−0.98 (−2.57,0.60)	−0.11 (−0.22,0.01)

Linear regression models, unstandardized coefficients.

95% confidence intervals in parentheses.

Significant associations in bold.

^*^ *p* < 0.05, ^**^ *p* < 0.01, ^***^ *p* < 0.001.

^a^Z-score of the sum of all cognitive tests (mean: 0, standard deviation: 1).

^b^Test-specific raw scores for each cognitive tests (see Methods section for further details). MMS: range (5, 12); Digit Symbol: range (2, 22); KOLT: range (6, 60); COWAT: range (3, 34); TMA: range (-154,-14; reversed); Block Design: range (2, 16).

^c^N=344.

^d^N=324.

^e^Mean of the interspinous distance, the intercristal distance and the external conjugate in centimeters.

**Table 4:** **Associations between dichotomous familial risk factors at birth and continuous cognitive outcomes at age 72–74 years (N=346)**

Exposures	Level of adjustment	Composite score^a^	Separate cognitive tests^b^					
			MMS	Digit symbol	KOLT	COWAT	TMA	Block design
Mother’s condition, good (vs poor)								
	Unadjusted	0.17 (−0.17,0.51)	0.09 (−0.21,0.40)	0.83 (−0.72,2.39)	0.74 (−2.05,3.53)	**2.26** ^*^ **(0.35,4.16)**	−2.27 (−12.61,8.06)	−0.25 (−0.99,0.49)
	+ age/gender	0.22 (−0.12,0.56)	0.13 (−0.18,0.43)	0.99 (−0.58,2.57)	1.20 (−1.55,3.96)	**2.39** ^*^ **(0.45,4.33**)	−1.53 (−12.02,8.95)	−0.21 (−0.96,0.55)
Family history of TB, no (vs yes)								
	Unadjusted	−0.17 (−0.49,0.15)	−0.07 (−0.35,0.22)	−0.04 (−1.51,1.42)	−2.02 (−4.64,0.60)	−1.42 (−3.23,0.38)	−1.90 (−11.63,7.83)	0.04 (−0.66,0.74)
	+ age/gender	−0.16 (−0.48,0.15)	−0.07 (−0.35,0.22)	−0.03 (−1.50,1.44)	−1.89 (−4.43,0.66)	−1.41 (−3.22,0.40)	−1.90 (−11.63,7.83)	0.04 (−0.66,0.74)
CVD, family, no (vs yes)								
	Unadjusted	−0.09 (−0.39,0.21)	−0.10 (−0.37,0.16)	−0.12 (−1.50,1.25)	−0.71 (−3.18,1.76)	0.35 (−1.35,2.05)	−6.82 (−15.94,2.29)	0.09 (−0.57,0.75)
	+ age/gender	−0.08 (−0.38,0.22)	−0.09 (−0.36,0.18)	−0.04 (−1.43,1.35)	−1.12 (−3.54,1.30)	0.34 (−1.39,2.06)	−6.04 (−15.26,3.17)	0.15 (−0.52,0.81)
Mother’s appearance, good (vs poor)								
	Unadjusted	0.13 (−0.10,0.37)	−0.09 (−0.30,0.12)	1.06 (−0.02,2.15)	0.52 (−1.44,2.47)	0.65 (−0.70,1.99)	4.76 (−2.46,11.97)	0.11 (−0.41,0.63)
	+ age/gender	0.12 (−0.11,0.36)	−0.10 (−0.31,0.11)	1.03 (−0.06,2.11)	0.51 (−1.40,2.41)	0.64 (−0.71,1.99)	4.44 (−2.80,11.68)	0.09 (−0.43,0.61)
Complications, no (vs yes)^c^								
	Unadjusted	0.25 (−0.07,0.58)	−0.12 (−0.42,0.17)	1.23 (−0.28,2.74)	1.11 (−1.62,3.83)	0.13 (−1.74,2.00)	9.92 (−0.10,19.94)	**0.88** ^*^ **(0.16,1.60)**
	+ age/gender	0.28 (−0.05,0.60)	−0.11 (−0.41,0.19)	1.31 (−0.21,2.83)	1.18 (−1.47,3.83)	0.15 (−1.74,2.03)	10.49^*^ (0.44,20.54)	**0.92** ^*^ **(0.20,1.64)**
Socioeconomic status, higher (vs lower)								
	Unadjusted	**0.25** ^*^ **(0.05,0.45)**	0.12(−0.06,0.30)	**1.16** ^*^ **(0.23,2.08)**	1.28 (−0.39,2.94)	0.72 (−0.43,1.87)	**6.78** ^*^ **(0.63,12.93)**	0.08 (−0.36,0.53)
	+ age/gender	**0.25** ^*^ **(0.05,0.45)**	0.12 (−0.06,0.30)	**1.16** ^*^ **(0.23,2.08)**	1.24 (−0.38,2.87)	0.72 (−0.43,1.87)	**6.82** ^*^ **(0.66,12.97)**	0.09 (−0.36,0.53)
Unmarried, no (vs yes)								
	Unadjusted	−0.06 (−0.59,0.46)	−0.06 (−0.54,0.41)	−0.73 (−3.17,1.70)	−1.33 (−5.70,3.04)	2.68 (−0.31,5.68)	2.32 (−13.85,18.50)	−0.87 (−2.02,0.29)
	+ age/gender	−0.12 (−0.65,0.41)	−0.10 (−0.57,0.38)	−0.88 (−3.34,1.57)	−1.88 (−6.16,2.39)	2.65 (−0.38,5.67)	1.56 (−14.73,17.86)	−0.93 (−2.09,0.24)
Teeth, lower jaw, good (vs poor)^d^								
	Unadjusted	0.06 (−0.14,0.26)	−0.09 (−0.26,0.09)	0.24 (−0.71,1.20)	**2.25** ^**^ **(0.56,3.93)**	0.10 (−1.08,1.28)	−0.78 (−6.96,5.40)	0.06 (−0.40,0.52)
	+ age/gender	0.09 (−0.11,0.29)	−0.08 (−0.25,0.10)	0.29 (−0.67,1.25)	**2.75** ^**^ **(1.11,4.39)**	0.15 (−1.04,1.35)	−0.70 (−6.92,5.53)	0.06 (−0.40,0.52)
Type of payment, insurance (vs other)^e^								
	Unadjusted	0.15 (−0.10,0.39)	0.17 (−0.07,0.41)	**1.33** ^*^ **(0.22,2.44)**	0.38 (−1.56,2.32)	−0.30 (−1.63,1.02)	−0.29 (−7.44,6.86)	0.07 (−0.45,0.59)
	+ age/gender	0.14 (−0.11,0.39)	0.17 (−0.07,0.41)	**1.30** ^*^ **(0.19,2.41)**	0.30 (−1.61,2.20)	−0.32 (−1.65,1.01)	−0.31 (−7.48,6.86)	0.06 (−0.46,0.59)
Number of diseases, ≤1 (vs >1)								
	Unadjusted	0.00 (−0.22,0.22)	−0.07 (−0.27,0.12)	0.42 (−0.58,1.43)	−0.53 (−2.33,1.27)	0.32 (−0.92,1.56)	1.91 (−4.77,8.58)	−0.12 (−0.60,0.36)
	+ age/gender	−0.01 (−0.23,0.21)	−0.08 (−0.27,0.12)	0.39 (−0.62,1.40)	−0.54 (−2.29,1.22)	0.32 (−0.93,1.56)	1.63 (−5.06,8.32)	−0.14 (−0.62,0.34)

Linear regression models, unstandardized coefficients.

95% confidence intervals in parentheses.

Significant associations in bold.

^*^ *p* < 0.05, ^**^ *p* < 0.01, ^***^ *p* < 0.001.

^a^Z-score of the sum of all cognitive tests (mean: 0, standard deviation: 1).

^b^Test-specific raw scores for each cognitive tests (see Methods section for further details). MMS: range (5, 12); Digit Symbol: range (2, 22); KOLT: range (6, 60); COWAT: range (3, 34); TMA: range (-154,-14; reversed); Block Design: range (2, 16).

^c^Including, but not limited to, prolonged labour, abnormal presentation assisted delivery of the baby (use of forceps) and episiotomy, uterine rupture, discoloured amniotic fluid, abnormal fetal souffle and placenta praevia, and combinations of these.

^d^N=335.

^e^N=245.

## Discussion

In this study investigating the association between the environment present around birth and cognition in old age, we found little evidence to support a substantial influence. We only found weak support for any anthropometric measures obtained at birth being predictive of cognitive function in old age. Specifically, only head circumference was associated with a better performance on COWAT and TMA in old age in the unadjusted model. This is contradictory to a previous paper which found no association between head circumference at birth and adult cognitive function, but a positive association between adult head circumference and adult cognitive function (Gale et al. 2003). Negative findings were present for birth complications and maternal health status. We did, however, find support for an association between higher parental SES (as measured by father’s occupation) and global cognitive function in old age in addition to specific associations with Digit Symbol and TMA test performance, both representing timed tests involving attention, speed and effortful mental processing. This highlights the importance of parental SES in relation to some specific domains of cognitive functioning in old age (Jefferis et al. 2002; Zhang et al. 2009), perhaps relatively independent of birth size (Zhang et al. 2009), a notion which was confirmed also in our study.

Important strengths of this study included access to birth records from the 1920s and the possibility to link this information to a population-based health survey in the late 1990s, enabling a 72–74 year follow-up. Data sources for both exposure and outcome status contained detailed information, and the gathering of information is unlikely to be biased in any particular direction. Considering the birth records, these were used at the time in the education of midwives under the supervision of the head physician with a high level of attention to quality, and included detailed anthropometric measures, as well as information about maternal health and circumstances, the birth process and the early post-natal period. Another strength, is that the cognitive examination part of the HUSK study included cognitive tests investigating several different cognitive domains ranging from episodic memory, executive function visuospatial and motor skills and verbal fluency.

A key limitation is that a relatively small proportion of the HUSK sample could be traced to their birth records. There are several potential reasons for this: the birth records were only available for a subgroup as not everyone who participated in HUSK was born in the Bergen area, and some were born at home or at other hospitals. Based on a conservative estimate, at least one-third of the HUSK sample would not be within the catchment area of the public maternity ward at the time of birth. The sample that was traced was representative of the participants in the HUSK cognitive examination. The results of the analysis are therefore likely to generalize to others of this generation and residence. However, it cannot be assumed that the traced participants were representative of people born in the location from which the early life records were taken. In particular, the representativeness of the birth cohort in HUSK might well have been influenced by intervening migration and survival effects because of the long follow-up. Healthy survivor effects (Baillargeon & Wilkinson 1999) or non-participation bias (Knudsen et al. 2010) are also possible. Negative findings could have resulted from inaccuracies in the measurement of either exposures or outcomes; for example, information on maternal health and family circumstances was derived from relatively crude measures. However, despite this, the similarly crude measure of parental SES included provided the most consistent significant associations identified with the outcomes. As previously described, three different social classes dominated Bergen during the time when the participants were born. Based on information from paternal occupational status, however, most of the participants in our study sample were from middle to lower socioeconomic strata with the occupation of the fathers varying from unskilled manual workers to teachers and general managers. This should be considered as a characteristic of the analysed sample when interpreting findings. Given the high number of associations tested, Type I errors cannot be ruled out, although we chose to focus on patterns of significant associations rather than significant associations per se. Differential bias arising from measurement is unlikely since reporting in HUSK is unlikely to be influenced by birth circumstances and recording of birth circumstances was carried out blind to all HUSK measures. The low traceability and small sample size constitute central limitations to our study, and warrants caution with regards to the precision of our estimates, and the interpretation and generalisability of the present study. Also of note, the limited size of the sample did not provide sufficient statistical power to specifically investigate low (<2.5 kg) or high (>4.5 kg) birth weight, or any influence of rare birth complications on cognitive function in old age, such as obstruction, foetal hypoxia or abnormally low birth weight.

### Interpretation of our findings

We found little evidence to support a substantial association between intrauterine or birth environment and cognitive function in old age in general. The only anthropometric measure which to a certain degree predicted cognition in old age was head circumference, and parental SES was the only exposure which was associated with the composite cognitive score. Both a higher head circumference and SES seemed to predict a higher cognitive function in old age independently of each other. One potential explanation for this is that early SES and head circumference are predictors of two different aspects of later cognitive function (Stern 2002). The association between SES and later cognitive function may represent cognitive reserve, while the association between head circumference and later cognitive function may represent brain reserve, both of which are relevant concepts for understanding cognitive function and vulnerability to cognitive impairments in old age (Stern 2002). In this respect, it also interesting that adjusting for educational attainment substantially weakened the associations between paternal SES and cognitive function in old age, suggesting that these associations might be substantially mediated through education. On the other hand, self-rated health reported in later life did not appear to influence these associations meaningfully. Further specific causal pathway modeling was felt to be beyond the scope of this study and not warranted by the largely negative associations of interest.

The lack of a substantial association between intrauterine or birth environment and cognitive function in old age, may be also be a reflection of a diminished impact of these early factors as other influences comes into play across the lifespan (Zhang et al. 2009). Both birth weight and socioeconomic status have been found to be associated with cognitive function in childhood (Shenkin et al. 2001; Jefferis et al. 2002), although socioeconomic status and postnatal influences have been suggested to be more important than prenatal factors (Jefferis et al. 2002; Erickson et al. 2010), similar to our own finding of the importance of parental SES. Other studies have also found that social disadvantage and early life stressors are related to cognitive function in later life (Mak et al. 2006; Nguyen et al. 2008; Fors et al. 2009), and it is generally accepted that childhood SES is an important predictor for later cognitive function (Mak et al. 2006; Hackman & Farah 2009), and cognitive reserve (Stern 2002). Even though anthropometric measures obtained at birth did not predict cognitive function later in life, it is possible that other factors mitigated these initial differences and reduced or eliminated their influence in later adult life (Zhang et al. 2009). These may include later nutrition, education and occupational status (Stern 2002).

## Conclusion

In conclusion, we found little evidence to support a substantial association between intrauterine or birth environment and cognitive function in old age in general. There were, however, some findings relating socioeconomic status and head circumference at birth and cognitive functioning in old age that warrants further investigation.
